# A combination of ultrasonic debridement and topical cortex phellodendri compound fluid in patients with diabetic foot ulcers

**DOI:** 10.1097/MD.0000000000029604

**Published:** 2022-08-12

**Authors:** Hang Gao, Jiali Chen, Ziying Zhao, Guangyi Wang

**Affiliations:** a Acupuncture Department, Master of Science, the Affiliated Hospital of Guizhou Medical University, Guizhou Province, China.

**Keywords:** cortex phellodendri compound fluid, diabetic foot ulcers, Kangfuxin liquid, traditional Chinese medicine, ultrasonic debridement

## Abstract

**Objective:**

To evaluate the combination of ultrasonic debridement and cortex phellodendri compound fluid (CPCF) in patients with diabetic foot ulcers (DFU).

**Patients and methods:**

Patients with DFU received the combination of ultrasonic debridement and CPCF in the experimental group and Kangfuxin liquid in the control group for 4 weeks. Patients total clinical efficiency, adverse events, ulcer areas, healing rate, and positive bacterial culture rate were compared.

**Results:**

The total clinical efficacy was 98% in the treatment group and 68% in the control group (*P* < .0001). Patients’ adverse events did not show significant difference between 2 groups. Patients who received the combination of ultrasonic debridement and CPCF in the experimental group had smaller ulcer areas (2.88 ± 0.2408 vs 6.912 ± 0.4044), higher healing rate (96.25 ± 0.5263 vs 55 ± 0.8888), and lower positive bacterial culture rate (0 vs 20%) than patients received Kangfuxin liquid in the control group after 4 weeks of treatment.

**Conclusions:**

In conclusion, patients with DFU receiving the combination of ultrasonic debridement and CPCF had better clinical efficacy, smaller ulcer areas, higher healing rate, and lower positive bacterial culture rate without increasing the adverse events compared to patients receiving Kangfuxin liquid.

## 1. Introduction

Diabetic foot ulcers (DFU) is a common complication that occurs in diabetic patients whose disease are poorly controlled, with an incidence of approximately 15%.^[1]^ A nonhealing of DFU might even cause severe damage to the bone and tissue and sometimes even amputation is needed.^[2]^ According to a meta-analysis, the prevalence of DFU is 5.5% in Asia.^[3]^ Diabetic patients had an amputation rate 7 to 10 times higher than other patients without diabetes.^[4]^

To treat and control the DFU, ultrasonic debridement can be used to remove bacterial, fungal, and dead tissue by penetrating into all parts of the wound cavity and hemostatic functions during jet flushing using the high-energy and low-frequency ultrasound waves.^[5]^ Ultrasonic debridement is used not only for controlling the infection, but also for controlling the DFU with the help of ultrasonic thermal and biological effects. The thermal effect can raise the skin temperature and thus help healing the ulcers. Herberger et al did a randomized controlled trial and proved ultrasonic debridement had the healing effect as good as surgical debridement in patients with DFU.^[6]^ Another study of 60 patients with DFU proved that patients receiving ultrasonic debridement had better mean clinical efficacy compared to patients in the control group.^[7]^

The traditional Chinese medicine (TCM) has long been used and has a significant role in the clinic for treatment of DFU. Among them, cortex phellodendri compound fluid (CPCF) can reduce the purulent secretions of wounds, reduce pain, promote inflammatory resolution of wounds, and thus could effectively promote wound healing in DFU treatment.^[8]^ CPCF consists of Cortex Phellodendri, Forsythia suspensa, Lonicera japonica Thunb, Taraxacum mongolicum Hand.-Mazz, and Scolopendra.^[8]^ Cortex Phellodendri is known to be one of the fundamental herbs of TCM. It is used to clear heat, reduce fire, dry dampness and release toxins.^[9]^ It has the antipathogenic microorganism and antiulcer function.^[10]^ Forsythia suspensa is widely and traditionally used to treat fever and inflammation as a TCM.^[11]^ Modern researches reveal its anti-inflammatory,^[12]^ antioxidant,^[13]^ antibacterial,^[14]^ and neuroprotective functions.^[15]^ Lonicera japonica Thunb is a common herb in East Asia with antioxidant properties.^[16]^ Taraxacum mongolicum Hand.-Mazz is a dietic herb for heat-clearing and detoxifying functions.^[17]^ Scolopendra also has anti-inflammatory effect. These ingredients of CPCF work together and might be helpful in the treatment of DFU. A study of 720 patients with DFU proved that after 4 weeks of treatment of CPCF, patients had reduced ulcer area and increased levels of growth factors, proving that it is more effective than the control solution.^[18]^ Another widely used TCM in the treatment of DFU is Kangfuxin liquid, which is a pure herbal extracted from the Periplaneta americana used in treatment of many ulcers disease.^[19]^ A meta-analysis of 11 trials involving 889 patients with DFU showed that Kangfuxin liquid combined with basic treatment could improve total effective rate (risk ratio = 1.38, 95% confidence interval [CI] = 1.23–1.54, *P* < .00001) and cure rate (risk ratio = 1.67, 95% CI = 1.17–2.38, *P* = .005).^[19]^ Thus, we decided to evaluate the combination of ultrasonic debridement and CPCF in patients with DFUs in comparison with Kangfuxin liquid in the control group. Our hypothesis is that patients treated with the combination of ultrasonic debridement and CPCF had better clinical efficiency without increasing the adverse events.

## 2. Patients and Method

### 2.1. Patients

One hundred patients diagnosed with diabetes with presence of DFU were recruited in our study between January 1, 2018, and December 31, 2019. Patients’ inclusion criteria are diagnosis of diabetes following the 1999 World Health Organization’s criteria,^[20]^ and patients met the diagnostic criteria of syndromes for DFU.^[21]^ We recruited patients only with an age of 35 to 70 years and ulcer area of <10 cm^2^. Patients with other complications, including coronary artery disease, kidney disease, liver disease, severe malnutrition, were excluded from our study. We also excluded patients with ulcers caused by radiation or other causes. Patients in the treatment group received the combination of ultrasonic debridement and CPCF for 4 weeks. Patients in the control group received Kangfuxin liquid for 4 weeks. The study was approved by the Ethics Committee of the affiliated hospital of Guizhou Medical University. All patients signed the written informed consent. We recorded patients’ baseline clinical characteristics, including age, sex, diabetes duration (years), number of patients with diabetic mixed ulcers, diabetic venous ulcer, diabetic ischemic ulcer, and patients’ Wagner grading.

### 2.2. Treatment

DFU, including its area, depth, infectious status, exudation, and ulcer base were evaluated and confirmed by 2 experienced trained doctors. Patients in the 2 groups received the same drugs to control blood glucose levels and infection.

Patients in the treatment group received ultrasonic debridement (machine model: MUI-IV- H/S; Tengyue, Chongqing, China). The machine include several functions, such as digital temperature control, ultrasonic debridement, negative pressure suction, and high-pressure flushing. Therapeutics accomplished the procedure according to standard regulations. Each patient received the ultrasonic debridement once a day for 15 to 20 minutes. After the ultrasonic debridement procedure, CPCF (Shandong Hanfang Pharmaceutical Co., 100 ml/bottle, code number approved by SFDA: Z10950097) was used to cover the ulcer skin, then doctors covered the ulcer with sterile gauze and bandaging. The dosage of CPCF was used according to instructions. For deeper DFU, at least 20 mL of CPCF was used to rinse the ulcer.

Patients in the control group received at least 20 mL of Kangfuxin liquid (Sichuan Good Doctor Panxi Pharmaceutical Co., 100 mL/bottle, code number approved by SFDA: Z51021834) to rinse the ulcer according to instruction. All patients finished 4 weeks of treatment without taking any other drugs to rinse the ulcer. Patients in both groups received a low-fat and low-salt diabetes diet. They were also asked to keep the wound clean.

### 2.3. Clinical efficiency observation

Patient’s total clinical efficiency was observed at both baseline and 4 weeks after treatment. Clinical efficacy was evaluated following the criteria as followed: invalid means—patient’s ulcer surface did not have chance or even became worse with a healing area surface of <40%; improved means—patients’ ulcer surface had shrunk, necrotic tissue was decreased, and purulent secretion had disappeared, with a healing rate of 40% to 80%; effective means—ulcer was greatly recovered, with new granulation tissue and a healing rate of 80% to 100%; and cured means—ulcer was totally recovered, with a healing rate of 100% and all regional symptoms disappeared. Cured rate equals to the percentage of number of patients cured divided by the total number of patients.

### 2.4. Ulcer area

The ulcer area was measured by the same clinician, who was blinded to the patient group. The ulcer shrinkage area equals to percentage of ulcer area decrease 4 weeks after treatment in comparison to baseline.

### 2.5. Bacterium cultures

We collected ulcer secretions for bacterium cultures at treatment baseline and 4 weeks after treatment. Percentage of bacterium cultures was calculated in 2 subgroups.

### 2.6. Adverse events observation

Adverse event data during the treatment were collected and recorded, including occasional hypoglycemia, ulcerative itching, or wound bleeding.

### 2.7. Statistical analysis

Patient baseline characteristics, comparisons of total clinical efficiency, adverse events, comparisons of the ulcer areas, healing rate, and the positive bacterial culture rate were compared using Mann–Whitney *U* test for categorical variables and Fisher exact test for continuous variables. Statistical significance was defined as *P* ≤ 0.05. All statistical analyses were performed using SPSS, version 16.0 (IBM Corporation, Armonk, NY).

## 3. Results

### 3.1. Baseline characteristics

One hundred patients were recruited for our study. Fifty patients were treated with the combination of ultrasonic debridement and CPCF and 50 patients were treated with Kangfuxin liquid in the control group. The other treatments were the same between 2 subgroups including diet, diabetic education, and treatment of decreasing blood glucose. Baseline clinical characteristics, including age, sex, diabetes duration (years), number of patients with diabetic mixed ulcers, diabetic venous ulcer, diabetic ischemic ulcer, and patients’ Wagner grading were recorded in 2 groups as shown in Table 1. In the experimental group, there were 27 males and 23 females, with a median age of 67 years old and diabetes duration of 16 years. Twenty-five patients had diabetic mixed ulcers, 15 patients had diabetic venous ulcer and 10 patients had diabetic ischemic ulcer. The number of patients in the Wagner grading I, II, and III were 6, 35, and 9, respectively. In the control group, there were 26 males and 24 females, with median age of 66 years and diabetes duration of 17 years. Twenty-five patients had diabetic mixed ulcers, 15 patients had diabetic venous ulcer, and 10 patients had diabetic ischemic ulcer. The number of patients in the Wagner grading I, II, and III were 7, 37, and 6, respectively.

**Table 1:** Patients’ baseline characteristics.

Characteristics	Experimental group	Control group
Number of patients	50	50
Sex		
Male	27	26
Female	23	24
Age (median age), yr	67	66
Diabetes duration (yr)	16	17
Diabetic mixed ulcers	25	25
Diabetic venous ulcer	15	15
Diabetic ischemic ulcer	10	10
Wagner grading		
I	6	7
II	35	37
III	9	6

### 3.2. Comparison of total clinical efficacy

The total clinical efficacy was 98% in the treatment group and 68% in the control group (*P* < .0001; Table 2).

**Table 2:** Comparison of total clinical efficacy.

Groups	Recovery (%)	Efficiency	Valid	Invalid	Total clinical efficacy	*P* value
Experimental	18 (36)	23 (46)	8 (16)	1 (2)	98%	<.0001
Control	6 (12)	19 (38%)	9 (18%)	16 (32)	68%	

### 3.3. Comparisons of adverse events

Patients’ adverse events did not have a significant difference between 2 groups, with both groups having 10% of total events (Table 3).

**Table 3:** Comparison of adverse events.

**Groups**	**Total events (%**)	**Hypoglycemia (%**)	**Pruritus on the ulcer surface (%**)	**Bleeding on the ulcer surface (%**)
Experimental	5 (10)	2 (4)	1 (2)	2 (4)
Control	5 (10)	2 (4)	2 (4)	1 (2)

### 3.4. Comparisons of ulcer areas

**Figure 1.**
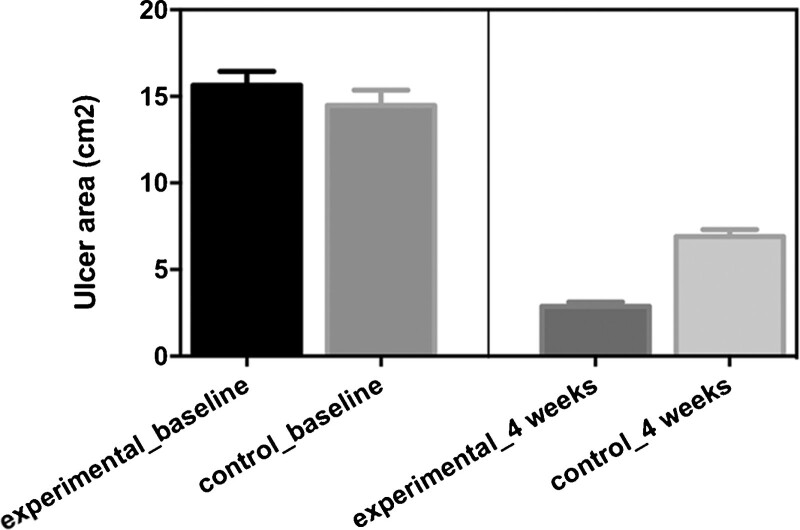
Comparisons of the ulcer areas between experimental group and control group.

Patients baseline ulcer areas between 2 groups did not have significant difference (15.64 ± 0.7935 vs 14.48 ± 0.8766, *P* = .3315; Fig. 1). Patients received the combination of ultrasonic debridement and CPCF in the experimental group had smaller ulcer areas than patients received Kangfuxin liquid in the control group after 4 weeks of treatment (2.88 ± 0.2408 vs 6.912 ± 0.4044, *P* < .0001; Fig. 1).

### 3.5. Comparisons of the healing rate

**Figure 2.**
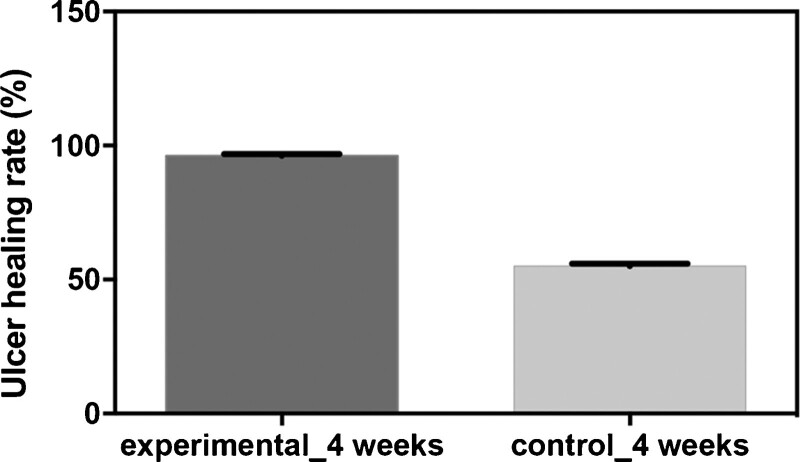
Comparisons of the healing rate between experimental group and control group.

Patients who received the combination of ultrasonic debridement and CPCF in the experimental group had higher healing rate than patients who received Kangfuxin liquid in the control group after 4 weeks of treatment (96.25 ± 0.5263 vs 55 ± 0.8888 *P* < .0001; Fig. 2).

### 3.6. Comparisons of the positive bacterial culture rate

**Figure 3.**
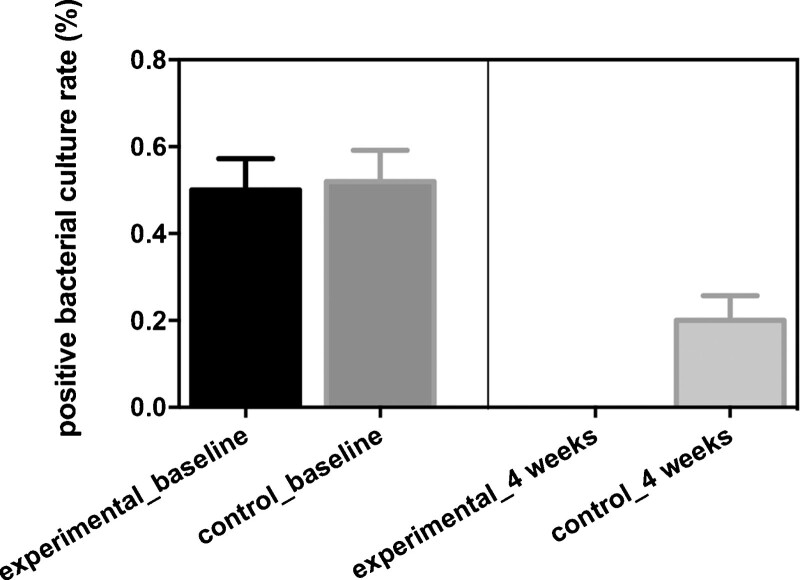
Comparisons of the positive bacterial culture rate between experimental group and control group.

Patients baseline positive bacterial culture rate between 2 groups did not have significant difference (50% vs 52%, *P* = .8434; Fig. 3). Patients who received the combination of ultrasonic debridement and CPCF in the experimental group had lower positive bacterial culture rate than patients who received Kangfuxin liquid in the control group after 4 weeks of treatment (0 vs 20%, *P* = .0007; Fig. 3).

## 4. Discussion

Our study was the first to prove that the combination of ultrasonic debridement and CPCF is effective and safe for treating DFU than the Kangfuxin liquid. Patients who received ultrasonic debridement and CPCF had better clinical efficacy, smaller ulcer areas, higher healing rate, and lower positive bacterial culture rate without increasing the adverse events compared to patients receiving Kangfuxin liquid. This indicated that a combination of ultrasonic debridement and TCM is a good treatment method for DFU.

Regarding the ultrasonic debridement, a previous study with 60 DFU patients has suggested that the combination of ultrasonic debridement with Shenghong wet dressing had significantly higher clinical efficacy than patients in the control group (93.33% vs 60.0%).^[7]^ Actually, ultrasonic debridement has been used in treatment of chronic slow or nonhealing ulcers.^[22]^ A randomized controlled trial proved that quality of life was observed to improve with the help of ultrasonic debridement as ulcers healed and pain levels reduced as ulcers improved.^[22]^ Another study of 46 patients with DFU receiving ultrasonic debridement accompanied together with standard wound care showed a beneficial effect on wound healing and wound infection.^[23]^ Our study reached the same results that DFU treated with ultrasonic debridement had smaller ulcer areas, higher healing rate, and lower positive bacterial culture rate than control group.

Ultrasonic debridement in combination with CPCF showed better clinical efficacy than standard treatment with Kangfuxin liquid, which indicated the significant function of TCM in the treatment of DFU. TCM has been long in the clinic in the treatment of DFU. Several TCMs have been used and proved to be clinically efficient. TCMs, including but not limited to Kangfuxin liquid, CPCF, Shenghong wet dressing, have been proved to be useful in healing the ulcers. A study proved that Chinese herbal medicine ulcer oil accelerates ulcer healing in a diabetic ulcer rat model, may downregulate levels of protein phosphotyrosine phosphatase 1B and advanced glycation end products, and upregulate vascular endothelial growth factor and platelet-derived growth factor, which may contribute to the inhibition of the inflammatory response and promote the healing of DFU.^[24]^ A meta-analysis suggested that TCM as an add-on treatment to traditional therapy could increase number of ulcer healings in patients with DFU.^[25]^ Another rat model of diabetic model study indicated that Shixiang plaster as TCM promotes healing of the ulcers through promoting angiogenesis.^[26]^ CPCF is a TCM that consists of multiple components with anti-inflammatory functions. The components of Cortex Phellodendri, Forsythia suspensa, Lonicera japonica Thunb, Taraxacum mongolicum Hand.-Mazz, and Scolopendra work together and achieve a better clinical efficacy and better ulcer healing rate for patients with DFU.

We must admit that our study also has limitations. First, our sample size is not large enough; thus, our results still need to be validated in a large clinical trial. We also did not investigate the mechanism that the combination of ultrasonic debridement with CPCF works better than traditional standard care. Ultrasonic debridement with CPCF might help reduce the inflammatory levels and promote the angiogenesis process. It is a very interesting aspect to further research the mechanisms of the combination of ultrasonic debridement with CPCF and better understand the healing process. Another limitation of our study is that there was no randomization and blinding in our study. However, our study was the first to come up with the idea that ultrasonic debridement and CPCF help patients with DFU a better healing and better clinical efficacy.

## 5. Conclusion

In conclusion, patients with DFU receiving the combination of ultrasonic debridement and CPCF had better clinical efficacy, smaller ulcer areas, higher healing rate, land ower positive bacterial culture rate without increasing the adverse events compared to patients receiving Kangfuxin liquid. The conclusion needs to be confirmed with more large-scale and well-designed clinical trials.
